# Food Supply Chain: A Framework for the Governance of Digital Traceability

**DOI:** 10.3390/foods14122032

**Published:** 2025-06-09

**Authors:** Maria Bonaria Lai, Daniele Vergamini, Gianluca Brunori

**Affiliations:** 1 Department of Economics and Business Sciences, University of Cagliari, 09123 Cagliari, Italy; mariab.lai@unica.it; 2 Department of Agriculture, Food and Environment, University of Pisa, 56124 Pisa, Italy; gianluca.brunori@unipi.it

**Keywords:** digitalization, business models, agrifood, traceability, value networks, blockchain, digital ledger technologies, supply chain, framework

## Abstract

Under the context of increasing demand for transparency, efficiency, and trust in food systems, digital traceability is emerging as a key strategy for improving value creation across agri-food supply chains. This study investigates how different governance structures influence the design and effectiveness of digital traceability systems. We develop an analytical framework linking four guiding questions (why, where, how, and who) to traceability performance and apply it to five Italian supply chains (wine, olive oil, cheese, pasta, and dairy) through 28 semi-structured interviews with companies, cooperatives, and technology providers. The results show that governance models shape traceability adoption and function. In captive systems (e.g., wine), traceability ensures compliance but limits flexibility, while in modular or relational systems (e.g., pasta and cheese), it fosters product differentiation and decentralized coordination. Across cases, digital traceability improved certification processes, enhanced consumer communication (e.g., via QR codes), and supported premium positioning. However, upstream–downstream integration remains weak, especially in agricultural stages, due to technical fragmentation and limited interoperability. The diverse experience data from company interviews reveal that only 30% of firms had fully integrated systems, and fixed costs remained largely unaffected, though variable cost reductions and quality improvements were reported in the olive oil and cheese sectors. The study concludes that digital traceability is not only a technical solution but a governance innovation whose success depends on the alignment between technology, actor roles, and institutional arrangements. Future research should explore consumer-side impacts and the role of public policy in fostering inclusive and effective traceability adoption.

## 1. Introduction

The agri-food sector is undergoing a profound transformation driven by the twin imperatives of digitalization and sustainability (Agri 4.0 refers to the digital transformation of agriculture through ICTs, enabling automation, data-driven planning, and interconnected value chains to improve efficiency, transparency, and sustainability [1]) [2,3]. Global challenges, including the projected 70% increase in food demand by 2050, climate change, and resource scarcity, are pushing agricultural systems toward more transparent, efficient, and resilient supply chains. In this context, digital traceability systems are increasingly recognized as essential tools to improve transparency, ensure regulatory compliance, and enhance value creation [3]. Digital traceability systems enhance transparency, compliance, and quality control across agri-food supply chains by enabling real-time monitoring of products [4,5]. Technologies like blockchain, IoT, and big data improve data integrity, prevent fraud, and support more sustainable and efficient resource management [1].

Despite rising academic and policy interest, current approaches often overlook the interplay between digital traceability and supply chain governance [6,7,8]. Most studies remain technology-centered, focusing on isolated innovations, such as Unmanned Aerial Vehicles (UAVs), the Internet of Things (IoT), and Radio Frequency Identification (RFID), without examining their systemic integration. As a result, enabling technologies like blockchain remain underutilized, particularly regarding their role in reinforcing transparency, data security, and decentralized control [9,10,11,12,13,14]. Moreover, the literature seldom investigates how different governance models, ranging from centralized consortia to loosely connected market structures, influence the design, adoption, and value distribution of traceability systems.

This paper addresses these gaps by asking: “What are the implications of different supply chain governance structures for digital traceability?” To answer this question, we develop and apply an analytical framework that links digital traceability functions with the organizational, technological, and institutional configurations of agri-food value networks. The framework is tested across multiple Italian’s agri-food supply chains (wine, olive oil, cheese, and pasta) through a literature review, semi-structured interviews, and empirical case studies.

The objective of the study is to investigate how digital traceability systems, particularly those enabled by blockchain and other distributed ledger technologies, can support value creation and equitable value distribution across agri-food supply chains. By focusing on governance dynamics and technological integration, the research aims to generate business model insights and operational guidelines for building more transparent, resilient, and innovation-oriented value networks.

The framework proposed in this study offers a novel contribution by systematically linking supply chain governance configurations with the design and performance of digital traceability systems, thus bridging two fields that are often treated separately in the literature.

By adopting a comparative and cross-sectoral approach, the study contributes to advancing current knowledge in two main ways. First, it sheds light on how different governance structures influence the design, implementation, and effectiveness of digital traceability systems. Second, it offers practical insights to support value creation and fairer value distribution within agri-food supply chains, with particular attention to the role and benefit of primary producers.

In doing so, the study addresses the international need for traceability solutions that go beyond compliance to promote trust, inclusivity, and sustainability in global agri-food systems. The remainder of this work is structured as follows: Section 2 presents the analytical framework that has been developed for the purpose of this research. This framework is based on the concepts of traceability, as proposed by Islam and Cullen, and the value network concept as defined by Porter. In Section 3, after a brief description of the sample, we analyze the interviewed companies selected for the use cases, grouping them into three main categories: primary producers, agri-food processing firms, and ICT providers. Finally, Section 4 is a discussion of our results and Section 5 contains our concluding remarks.

## 2. Materials and Methods

Traceability has developed unevenly across food supply chains, shaped by growing complexity and the need for efficiency, control, safety, and quality [15]. Islam and Cullen define it as *“the ability to access specific information about a food product that has been captured and integrated with the product’s recorded identification throughout the supply chain”*, a notion linked closely to operations such as tracking and tracing [16,17,18,19].

Today, traceability is strategic for managing value networks, ensuring regulatory compliance, enhancing transparency, and supporting integrated and secure data flows [20,21]. However, while Islam and Cullen’s framework offers a broad view of traceability within food systems, it is less suitable for analyzing its application within specific agri-food chains.

To fill this gap, our study develops an analytical framework to assess how digital traceability systems contribute to value creation and distribution. It addresses four core questions:

Why trace? How do governance structures influence traceability?

Where to trace? How does specialization affect system effectiveness?

How to trace? How does integration shape data collection and use?

Who is involved? How do actors’ roles affect a system’s design and impact?

Understanding value networks is essential to address these questions, as it allows for mapping how diverse stakeholders contribute to more transparent, efficient, and sustainable agri-food supply chains.

### 2.1. Analytical Framework

The concept of value networks builds on Porter’s foundational idea of value chains as sequences of activities creating value within organizations [22]. Later work has expanded this view, emphasizing the distributed and collaborative nature of value creation across interconnected actors throughout the product life cycle, from resource extraction to disposal [23,24,25,26].

In line with approaches to sustainability thinking, the term *value network* highlights the dynamic relationships among diverse stakeholders involved in the production, transformation, and distribution of goods and services [27]. In agri-food systems, this perspective shifts the focus from linear processes to complex, interconnected systems of actors, operations, and information flows [28,29,30].

Value networks are composed of three main elements: actors, operations, and linkages, in which flows of inputs, outputs, and information determine how value is created, shared, and sustained [31,32,33,34]. Their governance structures, ranging from market-based to modular, relational, and captive forms [24], shape the functioning of traceability systems and affect how value is distributed across the network.

**Figure 1:**
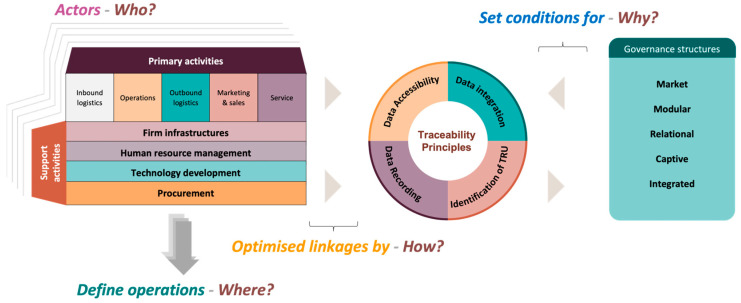
Analytical framework of value chain network traceability.

By integrating the traceability principles outlined by Islam and Cullen [15] (identification, data recording, integration, and accessibility), our framework helps to optimize the structure of value networks (Figure 1). Governance models play a central role in this process, as they can either enable or constrain the implementation of traceability systems, thereby influencing the overall efficiency, transparency, and distribution of value within agri-food supply chains.

In the following sections, we outline the analytical framework by illustrating how traceability systems relate to governance structures, operations, actor roles, and data flows within value networks.

#### 2.1.1. Why Trace? Governance Structures and Transaction Complexity

The rationale for adopting traceability systems varies across value network configurations and is closely tied to governance structures and transaction complexity. Drawing on Gereffi et al. [24], we distinguish between market, modular, relational, and captive governance (Gereffi et al. distinguish five governance types: *market* (low coordination, arms-length transactions), *modular* (standardized inputs and codified knowledge), *relational* (trust-based, frequent interactions), *captive* (small suppliers dependent on powerful buyers), and *hierarchical* (vertical integration under full control of the lead firm) [24]), each involving different levels of control and information asymmetry.

Mandatory traceability, driven by regulatory compliance for food safety and quality, is more common in captive and modular networks, in which lead firms manage complex transactions through standardized protocols and centralized platforms, though these may still entail risks of data asymmetry.

Voluntary traceability, by contrast, is a strategic choice in market and relational networks to foster transparency, trust, and product differentiation. In such settings, blockchain is increasingly used to support decentralized data management and enhance trust among autonomous actors [35].

Ultimately, the governance model shapes both the function and architecture of traceability systems within agri-food value networks.

#### 2.1.2. Where to Trace? Internal vs. External Operations and Specialization

The question of where to trace concerns the critical points along the supply chain for data collection and information management. Islam and Cullen [15] distinguish between *internal traceability*, within a single organization, and *external traceability*, which spans multiple actors from production to consumption.

Our framework highlights how organizational specialization influences traceability needs. Different functions (e.g., production, processing, and marketing) vary in technical capacity, access to digital tools, and incentives to collect data. Fieldwork has revealed that information is often concentrated in technically skilled units, creating asymmetries that hinder coordination and value sharing. Technical departments focus on process data, while commercial units prioritize certification and communication. Aligning traceability systems with the distinct needs of each unit and ensuring interoperability across functions are essential to identify critical control points and design effective, targeted systems in complex value chains.

#### 2.1.3. Who Traces? Actors, Responsibilities, and Governance of Information

The effectiveness of a traceability system depends not only on technology, but also on clearly defined roles and responsibilities. In mandatory systems, these roles are typically established by regulatory frameworks. In voluntary contexts, however, defining who collects, owns, verifies, and governs data becomes a core design challenge.

A robust traceability system must clarify: (i) who collects the data; (ii) who provides the technological infrastructure and services; (iii) who accesses and verifies the data; and (iv) who defines the rules and standards for data use. These functions unfold within a broader ecosystem involving producers, cooperatives, ICT providers, certifiers, and regulatory authorities. Our fieldwork confirms that unclear or fragmented responsibilities can disrupt information flows, lead to duplication, and undermine trust. Coordinated action, through negotiated agreements and shared governance, is thus essential to ensure participation, transparency, and legitimacy across the value network.

#### 2.1.4. How to Trace? Mechanisms of Data Capture, Integration, and Use

The final question (“How to trace?”) addresses the technical and organizational mechanisms for capturing, recording, integrating, and sharing data along the value chain. As defined by Olsen et al. [36], a traceability system encompasses “the totality of data and operations capable of maintaining desired information about a product and its components through all or part of its production and utilization chain”.

Core components of an effective system include the identification of Traceable Resource Units (TRUs), ranging from individual items to aggregates, through unique codes or symbols. Advanced digitalization may involve Digital Twins, which link physical products to their complete data history via technologies such as IoT, cloud computing, blockchain, and AI.

Accurate and timely data recording is essential at each stage, covering origin, processing, and movement. This requires consistent documentation to ensure traceability integrity. Beyond internal use, data integration across organizations plays a key role in external traceability. This involves interoperable systems, standardized formats, and shared protocols, often supported by data lakes or data spaces, allowing for a coherent reconstruction of each TRU’s path.

Finally, data accessibility ensures that authorized actors can retrieve and use information efficiently and securely. Real-time data sharing improves responsiveness but also raises concerns about privacy and control. Managing access rights and encryption is therefore critical to balance transparency with data protection.

As Olsen and Borit [36] note, access to product information throughout its life cycle, from its origin to its transformation and distribution, is fundamental. In our framework, effective traceability depends not only on technical infrastructure and standardization, but also on trust, organizational capacity, and governance arrangements that enable reliable, usable, and value-generating systems.

### 2.2. Data Collection

To investigate how digital traceability systems function across different governance settings, the study adopted a qualitative, multi-step methodology designed to combine theoretical grounding with empirical insight. The analysis followed a three-step approach: (1) a literature review, (2) in-depth semi-structured interviews, and (3) an ex-post reflexive synthesis to develop and interpret use cases through the proposed analytical framework. The literature review aimed to identify key theoretical contributions to traceability, digitalization, and value chains, guided by the following question: “What are the most frequently used fundamental theoretical concepts in traceability systems?” Searches were conducted via Google Scholar and Scopus using combinations of terms such as “traceability”, “food”, “digitalisation”, “blockchain”, and “value chain”. Following an iterative review process, 50 relevant academic articles were selected, of which 6 were retained to support the development of the analytical framework [18,19].

Subsequently, the study conducted 28 in-depth semi-structured interviews to gather experiential data from actors across key agri-food supply chains (Table 1). The process began with 6 expert participants from the AGRITECH Centre, selected to ensure coverage of essential competencies (agri-food, IT, engineering, and legal). This was followed by the compilation of a broader list of 145 entities, including agri-food firms, ICT providers, and consultants, identified through project networks and public databases. From this pool, 28 companies that confirmed their availability were selected for interview. These covered the main supply chains analyzed in the study: cereals and pasta, olives and oil, wine, cow milk and dairy, and sheep milk and dairy. Additional interviews were conducted with ICT developers active across multiple sectors, contributing cross-cutting insights for use case development.

The interviews, carried out online between March 2023 and May 2024, combined an introductory frame with open-ended discussion. In some cases, company presentations preceded the Q&A. The interview protocol covered company context and strategies (8 questions), the traceability system (19 questions), and company description (4 questions). Managers or senior staff were interviewed to ensure information completeness, while the semi-structured format allowed for flexibility to explore emerging themes.

While no direct survey of consumers was conducted, some insights on consumer acceptance, trust, and perceived value of traceability tools (e.g., QR codes) were captured through the reflections and experience of producers and service providers. These statements reflect the interviewees’ perceptions of how digital traceability is received by end users and should be interpreted as such.

The final phase of data collection focused on developing five use cases from the interviewed companies and consortia (Table 2). All selected actors had implemented digital traceability systems and operated in different Italian regions, with varying levels of complexity and digital maturity. Cases were chosen to reflect strategic national supply chains (like wine) and a sufficient advancement of voluntary traceability practices, enabling meaningful analysis of value creation dynamics.

Each use case includes (i) a brief profile of the company and its traceability goals; (ii) a description of the supply chain; and (iii) an analysis structured around the four guiding questions of the analytical framework: who, where, how, and why.

Sectors in which the role of digital traceability, particularly in its voluntary forms, was not sufficiently developed or clearly defined, such as meat, were deliberately excluded to ensure analytical depth and comparability across cases.

## 3. Results

### 3.1. Actors: Who?

Several factors influence the successful implementation and effectiveness of digital traceability systems. Among these, the type of company, its economic and operational profile, market performance, and the structure of its network relations are key (Table 3).

The set of interviewed actors is sufficiently heterogeneous and, as noted in Section 2.2, includes not only agri-food firms but also related stakeholders. It comprises primary producers, both vertically integrated companies (e.g., wine and olive oil producers with their own mills) and cooperatives, as well as processing firms, certification bodies, consulting companies, and ICT providers engaged in promoting traceability technologies in the agri-food sector.

In the following paragraphs, we analyze the interviewed companies selected for the use cases, grouping them into three main categories: primary producers, agri-food processing firms, and ICT providers. The analysis also considers the specific characteristics of the supply chains in which these actors operate.

#### 3.1.1. Primary Producers (P,C)

Among primary producers, both vertically integrated firms and cooperatives, centralized decision making emerges as a key governance feature that facilitates the alignment between production choices and market strategies. For example, in the wine sector, cooperatives coordinate vineyard practices to ensure consistent quality and brand identity, while in the olive oil sector, vertically integrated firms leverage control over milling and bottling to reinforce product differentiation. In more developed companies, this governance model supports brand expansion and sustainability innovation; in less mature firms, the focus remains on quality assurance and the use of traceability to regain market competitiveness. However, the capacity to invest in digital traceability systems is strongly influenced by financial performance. Financially solid firms (e.g., P1, P6, C1, C2, and C5) are more likely to adopt digital tools strategically enhancing transparency, consumer trust, and product valorization. Conversely, firms in transition or financial distress (e.g., P3 and C4) may adopt traceability primarily to improve internal efficiency and restore performance.

Cooperatives generally show better financial stability, but their multilevel governance adds complexity: member suppliers may resist certain technologies, limiting implementation. The same applies to individual producers, who face investment barriers and lack coordination. As highlighted by C1, even when the benefits are recognized, the costs of adoption remain a constraint (e.g., 75,000 EUR over two years for software implementation).

#### 3.1.2. Agri-Food Industry (T)

Among the processing companies (Group T), including producers of artisanal chips, cheese, PGI pasta, and olive oil, the adoption of digital traceability largely depends on financial capacity and market orientation. T5, with a high ROE (34.99%), is positioned to lead in technological innovation, while T1 may require restructuring before pursuing similar investments. T2, which is financially stable, sees traceability as a lever for competitiveness.

Unlike primary producers, these companies typically have centralized governance, which allows for more agile and strategic decision making. This enables a closer alignment between commercial goals and technological innovation, especially when quality certifications and market differentiation are priorities. For example, T2 uses traceability to ensure cheese certification, while T3 leverages it to justify premium pricing and appeal to quality-sensitive consumers.

Traceability technologies, particularly the IoT and distributed ledgers, support production optimization, origin verification, and brand positioning. T1 and T3 benefit from operational efficiencies and certification, while T2 and T5 focus on improving product quality and differentiation. In companies with centralized governance and sufficient resources, traceability tends to be more readily integrated into operational and marketing strategies, supporting efforts within product differentiation and quality assurance.

#### 3.1.3. ICT Companies (ICT)

ICT companies play a strategic role in promoting digital traceability through the development and deployment of technologies such as the IoT and blockchain. Their objectives range from market expansion to showcasing innovation capacity, often through partnerships and pilot projects in the agri-food sector.

Financial performance is highly variable. Firms like ICT2 and ICT3, with strong ROE and ROA values, are well positioned for growth and leadership, while others, like ICT1 and ICT4, face financial constraints. For these firms, promoting traceability solutions can also serve internal goals, such as cost reduction and improved process control.

Across these cases, traceability is both a product and a business strategy. ICT firms demonstrate its value to clients through performance metrics (e.g., data security, operational efficiency, and certification readiness) and support adoption by offering technical integration and consultancy services. Their role is not only technological but also relational: by aligning their solutions with client needs, they help to shape decision-making processes within agri-food companies. ICT companies often act as enablers of innovation, positioning themselves at the intersection of technology and governance by supporting the integration of traceability solutions into client systems and demonstrating their strategic value in concrete operational contexts.

### 3.2. Define Operations: Where?

To address the second research question (”How does specialization in certain stages of the supply chain influence the effectiveness of digital traceability?”), we analyzed how digital tools are integrated across specific operational stages in four agri-food supply chains: wine, olive oil, pasta, and cheese. Our focus is on how traceability technologies interact with key primary and support activities, shaping value creation and capture dynamics. The analysis emphasizes ‘where’: the exact points in the process at which traceability generates operational, informational, or strategic impacts.

#### 3.2.1. Wine Supply Chain

In the wine supply chain, the adoption of digital traceability systems is primarily concentrated in the processing, packaging, bottling, and certification stages. These phases represent critical points at which traceability supports operational control, product standardization, and compliance with market and regulatory requirements, such as origin certifications (e.g., DOP) and organic labeling (Table 4).

The integration of technologies such as barcodes, QR codes, IoT systems, and digital management platforms (e.g., Dioniso) enables companies to track production flows, reduce processing errors, and enhance transparency for both consumers and regulatory authorities. In particular, QR codes allow producers to differentiate their products and improve communications on their sustainability and quality attributes to the market. These findings align with the literature on digital traceability in wine supply chains, which highlights the sector’s strategic use of digital tools for quality assurance and competitive positioning in mature markets [35].

*“Digital traceability helps client companies comply with food safety regulations and organic certifications, simplifying our task of ensuring compliance”*

[CC1]

*“The software investment cost around €75,000, amortized over two years”*

[C1]

In terms of support activities, companies have adopted cloud platforms, interoperable systems, and consultancy-based training to ensure implementation and operational continuity. However, one of the main barriers observed is the lack of interoperability between mandatory and voluntary systems, which leads to duplicated workflows and increased operational burdens. This issue is particularly relevant in cooperatives, in which the involvement of multiple member suppliers adds complexity and may hinder uniform adoption.

The strategic impact of digital traceability is also visible in marketing, in which enhanced product transparency strengthens consumer trust, improves brand positioning, and facilitates access to premium markets.

*“The company could develop continuous monitoring and advanced reporting services based on data collected from IoT and Blockchain technologies, adding value for clients”*

[CC1]

While the downstream integration of digital tools is relatively advanced, upstream agricultural data (e.g., vineyard conditions and harvest timing) remain underutilized. Several interviewees noted that better coordination between agricultural and transformation phases, enabled by interoperable platforms, could improve product quality, planning, and resource efficiency. This is particularly relevant in the context of climate variability, in which the ability to anticipate and adapt production processes becomes a strategic competency.

The analysis shows that traceability practices in the wine sector are most developed in the post-harvest and commercialization phases, during which companies make efforts to enhance control, compliance, and visibility (Table 4). At the same time, persistent issues such as limited interoperability between systems and weak integration with upstream agricultural data emerge as key constraints affecting broader implementation

#### 3.2.2. Olive Oil Supply Chain

In the olive oil sector, digital traceability systems are primarily integrated into the processing and packaging phases, with limited, though growing, application in field-level monitoring (Table 5). The focus is on ensuring compliance with quality certifications (e.g., PDO and organic), improving production documentation, and strengthening transparency for consumers and regulators. In this case, the “where” in regards to traceability is closely tied to the post-harvest stages, when technological infrastructure is more easily deployed and when value is most visibly captured through quality differentiation and market positioning.

Companies use a combination of blockchain systems, QR codes, and official traceability platforms such as the National Agricultural Information System (SIAN), with some also experimenting with field sensors and IoT devices. These tools enable the certification of origin and production processes, enhance documentation, and support real-time audits and compliance reporting.

*“These tools allow companies to meet customer demands for detailed certifications of individual batches, which standard organic certification alone could not satisfy”*

[P3]

*“Blockchain was adopted to reinforce transparency and product distinctiveness in a highly competitive niche market”*

[A|1]

Several companies emphasized the marketing potential of digital traceability, particularly when integrated with QR codes on product labels. This facilitates direct consumer access to production data and reinforces the brand’s value proposition. However, as some interviews noted, conveying the added value to consumers still requires targeted communication efforts and may not immediately translate into higher margins.

*“A bottle left on the shelf without adequate explanation was not appreciated by consumers, even if accompanied by a tag”*

[P1]

From an operational standpoint, the combination of internal servers, cloud platforms, and blockchain technologies allows for secure data storage and traceability management across multiple stages, from harvest to bottling. Nevertheless, several companies highlighted the limited interoperability between internal systems and external platforms, as well as the need for stronger coordination between the agricultural and processing stages. This misalignment often reduces the effectiveness of traceability in optimizing quality and efficiency throughout the chain.

The technical development of traceability remains uneven. Some companies have discontinued certain field sensors due to inefficiencies, while others are actively exploring the use of drones, real-time monitoring systems, and advanced data analytics to better forecast and control production variables. Across all cases, the availability of skilled personnel and ongoing training emerge as prerequisites for maximizing the potential of digital tools.

The analysis highlights that in the olive oil sector, traceability efforts are mostly concentrated after harvest, especially during processing, packaging, and certification. While digitalization improves operational control and marketing potential, limited system interoperability and a gap between agricultural and processing data flows remain key issues affecting full integration. Strengthening upstream–downstream coordination and investing in workforce competencies appear to be central to enhancing the role of traceability along the chain.

#### 3.2.3. Pasta and Cheese Supply Chain

In the pasta and cheese supply chains, digital traceability tools are mainly applied to monitor and document transformation processes, from raw material sourcing (wheat or milk) to final packaging (Table 6). Traceability is particularly relevant in ensuring compliance with origin and quality standards, especially for certified products like durum wheat pasta or PDO cheeses. In both sectors, the “where” in regards to traceability corresponds to the processing and post-processing phases, although some companies are investing in upstream integration through digital agriculture tools and seed tracking.

*“We identified the most suitable area for durum wheat production and established supply chain contracts with farmers. A certified product becomes a strong competitive lever”*

[T3]

*“The XFarm platform integrates crop and field data with processing information, building a full traceability history”*

[T4]

In the cheese sector, the blockchain ensures full traceability from milk collection to final product labeling. In some cases, systems also allow for dispute resolution and third-party verification through roles like the “insurer”, enhancing data integrity and supply chain trust.

*“Once the product is ready for sale, a QR code is activated, allowing consumers to verify all production steps”*

[ICT1]

*“Growing by Sharing, Sharing by Growing: sharing information increases perceived value and profitability across the supply chain”*

[ICT1]

Digital platforms are integrated with internal servers and cloud systems to manage and store data securely. While cheese producers tend to be more advanced in digitalizing transformation and certification, pasta producers are investing in traceability to link agricultural practices to the final product’s quality, particularly for high-value, certified products.

The adoption of advanced IoT systems and real-time monitoring remains partial. The need for technical skills, interoperable platforms, and coordination between farming and processing stages emerges as a common theme. Improved planning, quality assurance, and cost efficiency are among the expected benefits, provided companies can develop the necessary digital competencies and collaborative models.

### 3.3. Optimised Linkages—How?

To address the third research question (“What are the consequences of varying degrees of relationships between supply chain stages, and how do they affect the information collected and stored through different technologies?”), we focus on the interplay between technological infrastructure and organizational integration. The effectiveness of digital traceability depends not only on the tools adopted but also on the strength and quality of relationships between the actors and stages of the supply chain.

Drawing from five representative use cases, we assess how the level of coordination and communication affects four key dimensions of the traceability systems identified in the analytical framework: (a) identification of traceable resource units (TRUs), (b) data recording methods, (c) data integration across supply chain stages, and (d) the accessibility of traceability information to different stakeholders.

The analysis highlights how high levels of interdependence and collaborative governance contribute to better data flow, accuracy, and usability. Conversely, fragmented systems and weak relationships often result in duplicated efforts, low data reliability, and limited access. The following subsections provide a comparative overview across four supply chains: wine, olive oil, cheese, and pasta. A more detailed breakdown of operations and the specific types of data tracked supply chain is provided in Appendix A.

#### 3.3.1. Wine Supply Chain (Case C5)

In the wine sector, traceability is centered on the post-harvest and packaging stages, supported by robust relationships between producers, consortia, and regulatory bodies. The system tracks products from grape batches and fermentation tanks to bottles, using QR codes and process IDs.

Data recording combines automated systems and manual inputs, primarily for compliance purposes. While some interoperability issues persist, strong institutional relationships allow for relatively effective integration and the standardization of traceability data. The level of accessibility is high for authorities but remains more restricted for other stakeholders, depending on their role and platform access (Table 7).

*“Digital identifiers and certification protocols allow us to trace every batch from vineyard to bottle. However, integration across systems remains a challenge”*

[C5]

#### 3.3.2. Olive Oil Supply Chain (P3)

In the olive oil sector, traceability extends from harvest to bottling, with TRUs including olive batches and final products. Data are initially collected manually, then digitized by an external service provider, introducing a potential source of delay and error. The level of integration is moderate, as information flows are managed across separate systems (Table 8). The relationships among producers, mills, and service providers are functional, but the outsourcing of data management limits internal control and full system interoperability.

*“Manual input is necessary at several stages before external digitalization. The system works, but it slows things down”*

[P3]

#### 3.3.3. Cheese Supply Chain (ICT1, ICT4)

Cheese supply chains exhibit multi-stage traceability, from milk collection to cheese processing and aging. TRUs are defined at both the batch and product levels. Data collection involves a mix of manual entry, IoT devices, and mobile apps, but integration remains low to moderate due to system fragmentation. The blockchain is partially used to secure data, but the level of accessibility is uneven: QR codes allow for consumer access, while encrypted information is reserved for authorized users. While relationships exist across the chain, manual data management hinders system cohesion and increases error risk (Table 9).

*“Blockchain helps ensure integrity, but manual entry is still widespread and slows down the process”*

[ICT4]

### 3.4. Set Conditions: Why?

To address the question “Why trace?” in digitally enabled agri-food supply chains, this section explores the relationship between governance structures and the effectiveness of traceability systems. Based on five use cases, we analyze how different governance configurations shape the conditions under which digital traceability is developed, adopted, and scaled. We consider five interrelated dimensions: transaction complexity, supplier autonomy, the role of data intermediaries, the capacity to codify transactions, and governance type (Table 10). These dimensions offer insights into how governance acts as a condition-setting mechanism for traceability performance.

Across the five cases, the transaction complexity is consistently high or moderate/high, reflecting the involvement of multiple actors, strict certification requirements, and the integration of digital systems such as the blockchain. However, the way this complexity is governed varies significantly, with important implications for traceability implementation.

In captive configurations, such as the wine supply chain (C5), centralized control by consortia ensures uniform standards and supports consistent data collection, making traceability effective for regulatory compliance. Yet, this control also reduces supplier autonomy and can limit flexibility in customizing digital tools. The presence of relational elements, such as collaboration among producers, helps mitigate rigidity and supports data flow, though access tends to remain asymmetric.

In modular systems, such as the pasta chain (T3), suppliers operate with greater autonomy and adopt standardized digital tools (e.g., the blockchain and QR codes). In principle, modular governance is well aligned with the requirements of traceability systems based on codified data and interoperable standards. However, without robust coordination mechanisms, modular systems often suffer from fragmented integration and variable accessibility, limiting the overall reliability of the traceability framework.

Relational governance elements, which are present in several cases (e.g., olive oil and cheese), facilitate trust-based coordination and knowledge sharing. These relationships are valuable for the co-design and adaptation of traceability solutions but tend to be difficult to scale and formalize without digital infrastructure and clear access rules.

In practice, most supply chains operate under hybrid governance arrangements, blending elements of captive, modular, and relational models. In these cases, traceability emerges as a negotiated outcome shaped by the balance between centralized enforcement and decentralized implementation. For example, in the cheese supply chain, digital traceability is structured around central platforms and certification bodies, yet depends heavily on cooperation among diverse actors. Similarly, in the olive oil sector, the integration of the blockchain is mediated by external mills and certifiers who act as critical intermediaries for data collection and validation.

The analytical lens developed in our framework thus helps move beyond a purely descriptive understanding of governance. By interpreting governance as a structural and relational configuration that sets the conditions for traceability, we show that the decision to adopt and invest in traceability is shaped not only by market or regulatory demands but also by the underlying organization of the supply chain. In captive systems, traceability ensures conformity and reputation; in modular systems, it enables interoperability and value differentiation; and in relational contexts, it supports trust and adaptive coordination.

Across the analyzed cases, the rationale behind the implementation of digital traceability appears to vary depending on the governance structure. In more centralized settings, traceability tends to support regulatory compliance and brand protection, while in modular or hybrid systems, it is more closely associated with value differentiation, data interoperability, or coordination needs. These patterns reflect how different governance configurations shape both the function and the perceived utility of traceability systems within agri-food supply chains.

### 3.5. Successful Value Networks

Building on the previous analysis, this section identifies the key attributes that support or hinder the success of digital traceability systems. While governance structures shape the framework for implementation, the actual effectiveness of these systems depends on a combination of enabling and limiting factors, such as capacity building, cost efficiency, market differentiation, and societal value (Table 11).

The analysis across the five use cases highlights a dual role for traceability: improving transparency externally and fostering internal competencies. As suggested by [34] and cited in [25], value capture depends on a firm’s ability to develop capabilities that are not easily replicable. Traceability contributes to this by enhancing process visibility and enabling the strategic use of data.

Capacity-related benefits, especially knowledge exchange, research, and education, are consistently cited as positive outcomes across all supply chains. These elements support continuous learning and collaborative innovation. In contrast, cost reduction is more limited: fixed costs remain largely unaffected, while notable reductions in variable costs are only reported in the olive oil case. This confirms that traceability systems tend to improve resilience and process management rather than cut expenses.

Market-related benefits are widespread. All cases report improved product differentiation, enhanced brand positioning, and stronger access to premium markets. Verified traceability supports certifications (e.g., organic and sustainability), which play a key role in consumer trust. For instance, in the pasta chain, the traceability of local wheat varieties helps certify the Italian origin of production, creating added value. Similarly, in the olive oil case, blockchain-supported traceability was key to accessing Japanese markets that required certified organic production.

Societal and environmental impacts are also notable. While farmer well-being is not always explicitly mentioned, positive effects such as reduced stress, better control over production, and improved community engagement are reported, particularly in the olive oil sector. Environmental benefits are perceived in all cases, pointing to the broader systemic value of traceability. Lastly, public funding emerges as a cross-cutting enabler. Government support is crucial for adoption, especially for smaller actors who might otherwise lack the resources to invest in digital systems. This confirms the importance of supportive policies in scaling traceability innovations across agri-food systems.

## 4. Discussion

*Governance structures and strategic orientation (Why?)*

This study confirms that governance configurations shape not only the technical adoption of digital traceability systems, but also their underlying rationale and perceived utility. In captive governance models, such as the wine supply chain, traceability is mainly used to ensure compliance with regulatory standards and safeguard the product’s reputation. The centralized control exercised by consortia enables uniform quality and robust certification, but often limits innovation and flexibility in the use of digital technologies.

By contrast, in modular and hybrid systems like pasta or cheese, traceability functions as an enabler of product differentiation and market access. The ability to codify transactions using tools such as the blockchain and QR codes supports interoperability and value communication, particularly in high-value or certified segments. However, modular settings may also suffer from fragmented data flows and coordination gaps if not supported by strong relational linkages or institutional anchors.

These findings align with the literature on global value chain governance [26] and expand it by demonstrating how governance types also influence the success conditions for traceability systems, especially in agri-food sectors in which both compliance and market trust are crucial.

*Operational specialization and integration points (Where?)*

The analysis shows that digital traceability tends to be concentrated in the downstream phases of processing, packaging, and certification across all supply chains. These are the points at which the product value is most clearly articulated and traceability has tangible impacts on compliance and consumer communication.

However, upstream integration, particularly in agricultural production, remains weak. Several firms reported poor interoperability between field data and transformation records, due to either technical fragmentation or organizational silos. This is particularly evident for olive oil and wine, whose agronomic variables have a significant influence on product quality but are poorly integrated into traceability systems.

In sectors like pasta, efforts to digitally monitor upstream activities (e.g., wheat sourcing and seed selection) show promise for building full-chain traceability, but current implementations are still partial. These gaps confirm the importance of aligning traceability design with functional specialization and of ensuring cross-phase interoperability through interoperable platforms and shared protocols [19,36,37].

*Data architecture and technological coordination (How?)*

Effective traceability requires more than the adoption of advanced technologies; it depends on the integration of those technologies across organizational units and supply chain stages. The study reveals that even when tools such as blockchain and IoT are deployed, their benefits are constrained by low automation, fragmented systems, and unclear responsibilities.

Data intermediaries, especially those managing blockchain platforms, play a crucial role in securing and validating traceability data. While these actors often ensure high levels of data integrity and standardization, their presence also introduces dependency risks and can restrict stakeholder access. In most cases, consumer access remains limited to surface-level information via QR codes, while core traceability records are encrypted and held by a few intermediaries. This raises concerns about transparency and the actual empowerment of end-users.

Firms that manage to combine secure data flows with user-friendly transparency interfaces (e.g., QR-linked storytelling) demonstrate stronger market trust and differentiation. Yet, most interviewees emphasized the need for stronger coordination, better-trained staff, and improved interoperability to fully exploit the potential of digital tools.

Beyond technical challenges, digital traceability also raises important tensions between data ownership, privacy protection, and supply chain transparency. Several interviewees reported that some farmers were reluctant to share sensitive farming information, fearing reputational or competitive risks. These concerns highlight the need for clear data governance frameworks that define access rights, consent mechanisms, and accountability procedures, especially in systems relying on the blockchain or centralized platforms.

*Actor roles and institutional ecosystems (Who?)*

Across all cases, the role of key actors, such as primary producers, processors, and ICT providers, is central in shaping the design and impact of traceability systems. ICT firms act as enablers of innovation, but their influence depends on their ability to align technological solutions with client governance and market needs.

For cooperatives and vertically integrated firms, centralized decision making supports effective implementation but may reduce flexibility and bottom-up innovation. When governance is shared or negotiated, success often hinges on the clarity of roles and the establishment of shared protocols for data access and use.

In several cases, especially in the cheese and olive oil supply chains, certification bodies and technology providers act as data intermediaries and gatekeepers. This underscores the need for clearer institutional arrangements and governance mechanisms that define who owns, accesses, and governs traceability data.

A deeper reflection is needed on the structural barriers that hinder the widespread adoption of digital traceability systems, particularly among resource-constrained small- and medium-sized enterprises (SMEs). While our findings confirm that traceability can enhance transparency, compliance, and value creation, several systemic constraints persist. First, financial barriers remain a major concern. The interviewed companies reported that digital traceability systems, especially those integrating the IoT, blockchain, and platform services, often require upfront investments that exceed the budgetary capacity of SMEs. In some cases, implementation costs exceeded EUR 70,000 over two years, with limited short-term returns. Second, educational and skill-based constraints affect the capacity of SMEs to implement and manage digital tools effectively. Many smaller firms lack dedicated ICT staff and depend on external consultants or technology providers. This not only reduces the level of internal control but also limits their ability to adapt systems to changing operational needs. Third, infrastructural fragmentation, particularly in rural areas, impedes effective data integration and system interoperability. Limited broadband coverage and weak platform integration between agricultural and processing stages constrain the flow of information across the supply chain.

To address these challenges, the study suggests four actionable strategies: (1) promote shared digital infrastructures, such as cloud-based traceability platforms managed at the consortium or cooperative level, to reduce fixed costs and enhance interoperability; (2) develop targeted training programs co-designed by public institutions and industry actors to build digital literacy and reduce SMEs’ dependence on intermediaries; (3) implement public financial incentives, including tax credits, rural development grants, and result-based subsidies to support traceability adoption; and (4) foster inclusive governance mechanisms that simplify adoption paths for smaller actors, including modular systems and phased implementation models.

These recommendations aim to ensure that digital traceability does not become an exclusive solution for large enterprises but a shared lever for improving transparency, competitiveness, and sustainability across all segments of the agri-food sector.

*Strengths and Limitations*

This study contributes both theoretically and empirically to the understanding of digital traceability as a socio-technical system embedded in specific governance and operational contexts. It provides a framework that integrates concepts from value network theory, digital infrastructure, and supply chain governance.

Its main strengths lie in the comparative and cross-sectoral approach used, the integration of qualitative insights from real use cases, and the development of a coherent analytical framework linking “why”, “where”, “how”, and “who” to traceability performance.

However, the study also presents some limitations. First, it focuses on producer and service provider perspectives, without systematically analyzing consumer behavior or willingness to pay for digital traceability. Although the interviewees reported perceptions of improved consumer trust, these remain second-order observations. Secondly, cost–benefit analyses of specific technologies (e.g., the blockchain) were not available in all cases and remain anecdotal. Future research should integrate quantitative performance metrics and explore consumer-side impacts to complete the picture.

## 5. Conclusions

Digital traceability is not a one-size-fits-all solution; its effectiveness depends on how it is embedded in the governance, operational, and relational structures of agri-food supply chains. This study shows that different governance models shape not only the architecture of traceability systems, but also their strategic function: from compliance and risk management in captive networks to value differentiation and trust-building in modular and relational contexts.

Across all supply chains, digital traceability emerges as a lever for value creation and strategic positioning, more than for cost reduction. It supports certification, strengthens brand identity, and enhances resilience, particularly in contexts in which quality, transparency, and reputation are critical market drivers.

However, its full potential remains constrained by gaps in data integration, limited access for end-users, and weak coordination across upstream–downstream stages. These limitations call for future investments in digital infrastructure, governance protocols, and skills development, especially among producers.

Future research should aim to deepen the understanding of how different design choices in traceability systems influence consumer behavior, particularly in terms of their trust, the use of their information, and their willingness to pay. In parallel, there is a need to assess the long-term economic and environmental impacts of these systems across diverse supply chains, considering both direct and indirect effects on performance and sustainability. Furthermore, investigating how public policy and targeted incentives can support the equitable diffusion of traceability technologies, especially among small-scale actors, will be crucial to ensuring that digital transformation contributes to fairer and more transparent agri-food systems.

Digital traceability is not only a technological innovation, but also a governance innovation. Its evolution will depend on our ability to design systems that are transparent, inclusive, and adaptive to the complexity of agri-food networks.

## Figures and Tables

**Table 1:** Overview and sample characteristics of interviews.

ID	Type	Location	Supply Chain	Traceability		Technologies
				Int	Est	
A|1	Organic food supplier	Emilia Romagna	Olive oil	✓	✓	Blockchain
A|2	Meat supplier	Lombardy	Meat	✓	✓	Management software + e-commerce
C1	Cooperative	Tuscany	Wine	✓	✓	BI software + vinification software
C2	Cooperative	Tuscany	Wine	✓	✓	Management software + sensors
C3	Cooperative	Apulia	Olive oil	✓	✓	ERP
C4	Cooperative	Tuscany	Wine	✓	✓	ERP + temperature sensors
C5	Consortium	Veneto	Wine	✓	✓	Management software + APP
C6	Cooperative	Sardinia	Wine	✓	✓	Management software
C7	Consortium	Campania	Olive oil	✓	✓	Blockchain + management software
C8	Cooperative	Tuscany	Olive oil	✓	✓	Milling software
CC1	Certification company	Lazio	Various		✓	Datalogger + App + AR + VR + blockchain
E1	Consulting firm	Tuscany	Various			Scanner software + RFID systems
E2	Consulting firm	Pidemont	Various			Blockchain + AI + smart collars
ICT1	Tech. company	Sardinia	Dairy	✓	✓	APP + blockchain + sc
ICT2	Tech. company	Campania	Trust services			
ICT3	Tech. company	Aosta Valley	Various			LoRa + low-energy sensors + blockchain
ICT4	Tech. company	Sardinia	Various			
P1	Farm	Apulia	Olive oil	✓	✓	Blockchain
P2	Winery	Tuscany	Wine	✓	✓	2 in-house software programs
P3	Farm	Tuscany	Olive oil	✓	✓	Blockchain + sc
P4	Farm + mill	Campania	Olive oil	✓	✓	Blockchain + ERP
P5	Farm + mill	Sardinia	Olive oil	✓		Milling software + TAG NFC
P6	Farm	Sardinia	Olive oil	✓	✓	Excel + management software + GPS
T1	Snack factory	Sardinia	Snacks	✓	✓	-
T2	Dairy factor	Abruzzo	Cheeses	✓		IoT + blockchain + ERP
T3	Pasta factory	Campania	Pasta	✓	✓	Blockchain + sc
T4	Pasta factory	Sardinia	Pasta	✓		2 farming platforms + smart harvester
T5	Mill company	Tuscany	Olive oil	✓	✓	Management software

**Table 2:** Overview of use cases and related traceability systems.

ID	Traceability Object	Traceability Driver	Who Benefits
C5	Wine: from grapes to bottles	Compliance, quality, brand trust	Producers, regulators, consumers
ICT1	Milk to cheese (incl. aging)	Quality, compliance, transparency	Farmers, producers, consumers
ICT4	Cheese: raw milk to final product	Quality, compliance, transparency	Producers, processors, brand owners
P3	Olive oil: harvest to bottling	Certification (DOP, organic), transparency	Producers, consumers, certifiers
T3	Pasta: wheat cultivation to packaging	Quality, transparency, origin verification	Farmers, millers, producers, consumers

**Table 3:** Interviewed companies—Products traced and economic performance.

*ID*	Main Spec.	Product Traced	Revenue	Net Profit	Employee	*ROE*	*ROA*
			EUR *		Number	%	
A|1	Marketing	EVO oil	>70MLN	>50K	100	0.33	0.22
A|2	Marketing	Meat	>1MNL	[>1MLN]	8	n.s.	−143.14
C1	Prod./marketing	Wine	>5MLN	>20K	21	1.56	0.78
C2	Prod./marketing	Wine	>13ML	<1k	14	0	1.06
C3	Prod./marketing	Olive oil	>7MLN	>9K	3	4.36	1.45
C4	Prod./marketing	Wine	>2MLN	>15K	10	1.67	1.04
C5	Protection/prom.	Wine	>500K	>34K	10	6.34	1
C6	Production	Wine	>11MN	<1K	34	0	0.59
C7	Protection/prom.	EVO oil	>200K	[>10K]	-	n.s.	−159.42
C8	Prod./marketing	Olive oil	>300K	<1K	7	29.84	15.32
CC1	Quality controls	Various	>30MLN	>400K	217	19.44	4.22
E1	R&D	Several	>500K	>24K	6	12.61	8.14
E2	Traceability services	Alpine grazing	-	-	-	-	-
ICT1	Olive growing	EVO oil	>70K	>3K	0	65.19	11.32
ICT2	Prod./marketing	Wine	>16MLN	>1k	36	0	1.6
ICT3	Arable/Olive growing	EVO oil	>50K	[<1K]	4	−4.33	−2.18
ICT4	Milling	PDO olive oil	-	-	-	-	-
P1	Milling	EVO oil	-	-	-	-	-
P2	Prod./marketing	EVO oil	>1MLN	>6K	3	5.86	1.28
P3	Transformation	Artisanal chips	>2MLN	>1K	13	0.18	1.38
P4	Transformation	Cheese	>3MLN	>60K	21	5.97	5.42
P5	Transformation	PGI pasta	>5MLN	<1K	15	0.06	0.26
P6	Transformation	Pasta	-	-	-	-	-
T1	Milling	Olive oil	>300K	>79K	3	34.99	17.34
T2	Traceability techn.	Cheese		[>13k]	0	-	−4.22
T3	Trust services	Various	>20MLN	>3MLN	99	36.83	28.66
T4	IoT solutions	Pesto sauce	>40K	>13K	0	92.57	56.49
T5	IoT solutions		>70MLN	[>3MLN]	0	−1.5	−1.84

* negative value in brackets; n.s. not supported.

**Table 4:** Integration of digital traceability in the wine supply chain.

Activity	Digital Tools/Focus	Observed Impact
Operations	Grape-to-wine tracking; digital bottling systems; AR/VR for process monitoring	Improved efficiency, error reduction, certification reliability
Outbound and Marketing	QR codes and digital labels on bottles	Stronger brand identity, market transparency, access to premium segments
Support Infrastructure	Cloud platforms, ERP systems, sensors, external consultancy	Data integration, system reliability, knowledge transfer
Value Creation	Integration of traceability and certification systems	Quality enhancement, compliance, environmental alignment
Value Capture	Integration of traceability and certification systems	Improved customer trust, premium pricing opportunities, market differentiation

**Table 5:** Integration of digital traceability in the olive oil supply chain.

Activity	Digital Tools/Focus	Observed Impact
Operations	Blockchain and SIAN systems for harvest-to-bottling tracking	Enhanced compliance, documentation, certification integrity
Outbound and Marketing	QR codes and digital labels on bottles	Improved consumer transparency, brand differentiation
Support Infrastructure	Internal servers, cloud platforms, digital archives	Secure data management, audit readiness
Technical Development	IoT devices, field sensors, drones (partially adopted or under evaluation)	Potential improvements in planning and real-time quality monitoring
Value Creation	Traceability aligned with PDO/organic certifications	Stronger quality assurance, enhanced market readiness
Value Capture	Consumer-facing traceability via QR codes	Justified premium pricing, increased brand trust and visibility

**Table 6:** Integration of digital traceability in the pasta and cheese supply chains.

Activity	Digital Tools/Focus	Observed Impact
Operations	Blockchain tracks cheese and pasta production; QR codes connect to packaging	Full production traceability, certification compliance, consumer transparency
Outbound and Marketing	QR codes on cheese and pasta packaging	Enhanced brand reputation, product differentiation
Support Infrastructure	Blockchain-integrated servers, internal platforms, cloud-based storage	Secure data access and storage, audit readiness
Technical Development	IoT sensors for cattle monitoring and crop tracking (partial or planned)	Early-stage digital agriculture integration, improved planning
Value Creation	Standardized traceability for certified products	Stronger authenticity, quality assurance, and regulatory alignment
Value Capture	Consumer-facing storytelling through digital tools	Premium positioning, improved market perception, traceability as added value

**Table 7:** Traceability snapshot—C5 (wine).

Dimension	Observation
Identification	Bottles, grape batches, fermentation tanks; individual product-level granularity
Data Recording	Mixed methods: manual + automated; compliance-oriented
Integration	Moderate; hindered by partial interoperability
Accessibility	High for regulatory bodies; limited for other actors via cloud-based platforms

**Table 8:** Traceability snapshot—P3 (olive oil).

Dimension	Observation
Identification	Batches of olives by harvest time; bottles
Data Recording	Manual input; digitized by third party
Integration	Moderate; outsourced management, fragmented flow
Accessibility	Moderate; QR codes for consumers, limited access to detailed data

**Table 9:** Traceability snapshot—ICT1 and ICT4 (cheese).

Dimension	Observation
Identification	Milk batches, cheese units, aging batches
Data Recording	Manual entry + digital apps + IoT; not fully automated
Integration	Low to moderate; fragmentation between systems
Accessibility	QR codes for consumers; blockchain access controlled for internal stakeholders

**Table 10:** Digital traceability and governance structures.

ID	Supply Chain	Complexity of Transactions	Supplier Autonomy	Data Intermediaries	Governance Type
C5	Wine	High	Low	Integral; Central role of C5	Captive with relational elements
ICT1	Cheese	Moderate/high	Low	Integral with a central role	Mix of captive, modular, relational
ICT4	Cheese	Moderate/high	Low	Integral with a central role	Mix of captive and modular
P3	Olive oil	High	Low	Integral; Key role of external mill and certification bodies	Mix of captive and modular
T3	Pasta	Moderate/high	Moderate/low	Critical role (e.g., blockchain providers, certifiers)	Modular with captive elements

**Table 11:** Identified positive or negative attributes of successful digital traceability system.

ID	C5	ICT1	ICT4	P3	T3
**Increased capacity**					
Research/education	✓	✓	✓	✓	✓
Knowledge exchange	✓	✓	✓	✓	✓
**Reduced costs**					
Reduced variable cost	X	X	X	✓	X
Reduced fixed costs	X	X	X	X	X
Production resilience	✓	✓	✓	✓	✓
Financial resilience	-	-	-	-	-
**Product differentiation valued by market**					
Differentiation by traceability	✓	✓	✓	✓	✓
Enhance market access	✓	✓	✓	✓	✓
Enabling marketing opportunities	✓	✓	✓	✓	✓
Certification: organic	X	✓	✓	✓	✓
Certification: animal welfare	X	✓	✓	X	X
Certification: sustainability	✓	✓	✓	✓	✓
**Societal value and farmer well-being**					
Enjoyment/improved well-being for farmer	-	-	-	✓	-
Community engagement	✓	✓	✓	✓	✓
Environmental benefits as perceived by farmer	✓	✓	✓	✓	✓
Animal welfare benefits as perceived by farmer	X	✓	✓	X	X
Government finance for digitalization	✓	✓	✓	✓	✓
Other support	-	-	-	-	-

✓ Positive factor mentioned; X Negative factor mentioned; - Non-relevant factor or not mentioned.

## Data Availability

The datasets presented in this article are not readily available because the data are part of an ongoing study. Requests to access the datasets should be directed to the corresponding author.

## Abbreviations

The following abbreviations are used in this manuscript:

EVO	Extra-virgin olive oil
PDO	Protected Designation of Origin
PGI	Protected Geographical Indication
TRU	Traceable Resource Unit

## Appendix A

**Table A1:** Olive Oil Supply Chain.

Operation and Type of Data Traced			
Origin and Cultivation Data	**Farm records:** Details about specific plots, number of trees, geographic layout, and treatments applied	**Agricultural operations:** Pruning, fertilization, pest control and other agronomic practices	
Harvesting and Transportation	**Harvest details:** Date, type and quantity of olives harvested	**Transport documents:** Origin of olives, supplier, fields, and delivery notes to the mill	**Transportation data:** Vehicle details, capacity, transport conditions
Processing and Milling	**Processing stages:** Quantity of olives received, washing, milling and crushing, malaxation, extraction, storage, and filtration	**Milling log:** Date of milling, type of extraction, yield, and quality of the product (Chemical-physical tests)	**Oil storage:** Tank number, movements, labelling (barcodes) and weight records
Quality Control and Certification	**Quality control:** Accompanying documents, PDO/PGI certification, acidity, phenol content, peroxide levels, organoleptic analysis parameters.	**Certification details:** Compliance certificates, hygienic conditions of storage tanks	Analytical reports and panel test evaluations
Bottling and Labelling	**Bottling process:** Entry of oil from the mill, yield from each milling batch, caps and bottles used	**Labelling:** Bottle tracking number, label generation, batch number, QR codes for traceability	**Packaging data:** Quantity of bottles produced, bottle format, minimum shelf life
Distribution and Sales	**Sales data:** Details of product transfer to customers, online and offline sales tracking	**Transport and logistics:** Tracking shipment routes, transit times, temperature and humidity monitoring	**Warehouse management:** Product flow, customer orders, storage, and dispatch

**Table A2:** Wine Supply Chain.

Operation and Type of Data Traced			
Origin and Cultivation Data	**Field operations:** Agricultural activities, water resources, pesticides, crop protection, and other environmental data		
Harvesting and Transportation	**Grape harvest:** Harvesting period, geolocation of vineyards	**Grape batches:** Grape type, year, quantity, alcohol content	**Transportation data:** Vehicle details, capacity, transport conditions
Processing	**Crushing or destemming:** Date, operation number, client, grapes being unloaded, products obtained.	**Racking:** Date, operation number, product being unloaded, new wine, products obtained.	**Vinification and aging process:** Tank identification (barcode), quality checks during processing.
Quality Control and Certification	**Quality control:** Analytical tests, quality control parameters	**Certification:** Compliance with PDO/PGI and Organic regulations, chemical analysis, and certification	
Bottling and Labelling	**Bottling process:** Test and quality check during bottling step, batch number, production records	**Labelling:** Barcode tracking, label generation, lot number	**Packaging data:** Bottles, caps, cartons, labels used
Distribution and Sales	**Sales data:** Product transfer, customer tracking (QR code)	**Logistics:** Shipment tracking, temperature and humidity monitoring, transit times	

**Table A3:** Cheese Supply Chain.

Operation and Type of Data Traced			
Origin and Cultivation Data	**Crop protection:** pesticides, water resources	**IoT sensors in the field:** plant health, soil moisture	
Breeding and Milk production	**Diets of cows and sheep:** lactation, dry periods	**Milk production:** quantity and quality of milk	**IoT sensors in the barn:** tempera ture and humidity
Processing	Milk collection and pasteurization	Transformation into fresh cheeses and pecorino	**Identification of cheese forms:** taleggio, ricotta, pecorino
Packaging and Labelling	**Batch traceability:** milk batch, tank number, production date	**Certification details:** Compliance certificates, hygienic conditions of storage tanks	
Transport and Distribution	**Transportation data:** Vehicle details, capacity, transport conditions	**Distribution traceability:** wholesalers, retailers, final customers	

**Table A4:** Pasta Supply Chain.

Operation and Type of Data Traced			
Origin and Cultivation Data	**Agricultural operations:** Pesticides, fertilization, pest control, water resources		
Harvesting and Transportation	**Harvesting:** date, variety, field location	**Transport documents:** Origin, supplier, fields, and delivery notes to the mill	**Transportation data:** Vehicle details, capacity, transport conditions
Processing and Milling (Semolina production)	Processing date, Semolina batch number, Orders details from the pasta factory		
Pasta production	Processing conditions	Pasta batch number	
Packaging and Labelling	**Batch traceability:** tank number, production date	**Packaging:** quantity of cartons, labels	
Distribution	**Distribution traceability:** wholesalers, retailers, final customers	**Packaging:** quantity of cartons, labels	
